# The Immunology of Takotsubo Syndrome

**DOI:** 10.3389/fimmu.2023.1254011

**Published:** 2023-10-06

**Authors:** Kenji Rowel Q. Lim, Douglas L. Mann, Tsuneaki Kenzaka, Tomohiro Hayashi

**Affiliations:** ^1^ Division of Cardiology, Department of Medicine, Center for Cardiovascular Research, Washington University School of Medicine, St. Louis, MO, United States; ^2^ Division of Community Medicine and Career Development, Kobe University Graduate School of Medicine, Kobe, Japan; ^3^ Department of Internal Medicine, Hyogo Prefectural Tamba Medical Center, Tamba, Japan

**Keywords:** Takotsubo syndrome, stress-induced cardiomyopathy, acute cardiac injury, inflammation, catecholamine stress, animal models

## Abstract

Takotsubo syndrome (TTS) is a disorder characterized by transient cardiac dysfunction with ventricular regional wall motion abnormalities, primarily thought to be caused by the effects of a sudden catecholamine surge on the heart. Although the majority of patients exhibit prompt recovery of their cardiac dysfunction, TTS remains associated with increased mortality rates acutely and at long-term, and there is currently no cure for TTS. Inflammation has been shown to play a key role in determining outcomes in TTS patients, as well as in the early pathogenesis of the disorder. There are also cases of TTS patients that have been successfully treated with anti-inflammatory therapies, supporting the importance of the inflammatory response in TTS. In this article, we provide a comprehensive review of the available clinical and pre-clinical literature on the immune response in TTS, in an effort to not only better understand the pathophysiology of TTS but also to generate insights on the treatment of patients with this disorder.

## 1 Introduction

Takotsubo syndrome (TTS) or stress-induced cardiomyopathy is an acute condition characterized by transient cardiac dysfunction that is associated with regional wall motion abnormalities of the left and/or right ventricles (1–3). Apical ballooning of the left ventricle (LV) is the most common presentation of TTS, but such dilation could also manifest in other LV regions (3, 4). The clinical features of TTS share a high degree of similarity to those of an acute myocardial infarction, with the two often distinguished by the lack of obstructed coronary arteries in the former (although coronary artery disease is not considered an exclusion criterion for TTS under recent International Takotsubo Diagnostic criteria) (1). Myocardial necrosis/fibrosis is substantially milder or absent in TTS (5–8). In roughly two-thirds of patients, physical or emotional triggers precede the index TTS event (4, 9). Moreover, about 90% of all TTS patients are women, nearly 90% of which are older than 50 years of age (1, 4). Due to the prompt recovery of LV function in patients, TTS was initially thought to be a benign condition. It is now recognized that TTS is associated with worse outcomes acutely, with an in-hospital mortality rate of 4-5% (2, 10), and at long-term, with an all-cause mortality rate of 5.6% per patient-year in addition to the persistence of certain symptoms (4). Multi-center studies also showed that TTS has a recurrence rate of 4-5% (11, 12). There is currently no cure for TTS.

The specific mechanisms that cause TTS remain an area of active research. The adrenergic hypothesis is currently the most prevalent, which explains that the pathophysiology of TTS is primarily built upon the effects of a catecholamine surge on the heart (1–3). This is supported by multiple lines of evidence, including among others the ability of stress or administered catecholamines to trigger TTS (4, 13, 14), the presence of elevated plasma catecholamine levels in TTS patients (5), the occurrence of TTS in patients with pheochromocytoma (15, 16), and the induction of TTS-like cardiac phenotypes in animal models given high catecholamine doses (8, 17–20). It is proposed that catecholamines either cause direct myocardial injury or do so indirectly by dysregulating myocardial metabolism or calcium dynamics (2, 3). However, as a strong relationship between a catecholamine surge and the development of TTS has not yet been established in patients (21), it is likely that other mechanisms are contributing to the unique pathophysiology of TTS.

One of these potential mechanisms is inflammation. While its contribution towards the initiation of TTS is largely unknown, it is generally regarded that inflammation plays an important role in influencing the long-term outcomes of the disease (2, 3, 8, 22). For instance, recent studies on 385-407 TTS patients from the Alsace Takotsubo registry revealed that residual inflammation at discharge (as defined by high C-reactive protein [CRP] levels) was associated with lower LV ejection fraction (LVEF) and higher rates of cardiovascular mortality among patients at a median follow-up of 2 years (23, 24). On a different note, conditions that modulate the immune system such as sepsis or the administration of immune checkpoint inhibitors have also been associated with inducing TTS (25, 26). In an effort to help better understand the roles of the immune system in driving or shaping TTS pathophysiology, we provide here a critical exploration of the available clinical and pre-clinical literature on the subject, as well as identify possible areas for further study.

## 2 Clinical research

### 2.1 Inflammation-associated serum biomarkers in TTS patients

TTS patients typically have elevated levels of inflammation-associated markers in the blood, such as CRP and leukocyte count, at least on or close to admission. Correlations have been made between increases in either or both parameters and reduced LVEF, increased cardiovascular mortality, increased catecholamine levels, as well as to a particular TTS trigger type (i.e., secondary to physical activities, medical conditions, or procedures) (6, 22–24, 27–32). In one study, multivariate Cox regression analysis identified a CRP level greater than 19 mg/L at discharge to be an independent predictor of cardiac mortality and heart failure re-hospitalization in TTS patients (23). While these findings suggest that acute inflammation considerably impacts TTS outcomes in patients, data on the levels of both markers at extended follow-up are lacking.

On that note, the serial measurement of immune cytokines in the blood has been useful in characterizing TTS-associated inflammation. Increased cytokine levels were observed acutely in TTS (22, 29, 33), with some such as interleukin (IL)-6 and IL-10 determined to be predictors for long-term adverse events or mortality in patients (34). To get a better idea of the chronic inflammatory state in TTS, the prospective TERRIFIC study (NCT02897739) measured serum cytokine levels in patients within 2 weeks from their index event and at follow-up 5 months later (22). It was found that the pro-inflammatory cytokine IL-6 was significantly elevated in TTS patients compared to matched controls acutely and at follow-up; IL-6 levels were ~3 times higher at 5 months versus controls. The chemokine IL-8 was similarly elevated at 5 months in patients. Finally, the chemokine CXCL1 was elevated at both time points, but was only significantly different from controls acutely. Since the majority of measured cytokines in the study was either not affected (IL-1β, IL-10, IL-12p40, tumor necrosis factor-α, interferon-γ) or decreased at 5 months (MCP-1), generalized inflammation in TTS is likely minimal, at least systemically. This was supported by another report from the same group where they measured these serum cytokines in a TTS patient cohort after a median follow-up of 20 months, and a trend for increased levels versus controls was only observed for CXCL1 (35).

### 2.2 Myocardial and systemic inflammation in TTS patients

In terms of cardiac inflammation, insights have been gained from cardiac magnetic resonance (CMR) imaging. Increased myocardial edema was found acutely in TTS patients, as evaluated by T2-weighted or native T1 imaging (6, 7, 22, 36, 37). Some reports indicate that the edema was regional in nature, being higher in dysfunctional areas of the LV, whereas others found the edema to broadly affect the entire LV and right ventricle (RV). Myocardial edema does gradually resolve in patients, but it can take time to completely normalize. For instance, myocardial edema can persist for 3 months and even up to 20 months after the index event in TTS patients, albeit at lower levels compared to those found acutely (35, 36). Using CMR imaging enhanced with ultrasmall superparamagnetic particles of iron oxide (USPIO), which are preferentially phagocytosed by macrophages, a study showed that TTS patients had significantly increased T2* values post-USPIO acutely, which resolved 5 months later (22), suggesting that inflammation in TTS could be macrophage-driven.

Macrophage infiltration in TTS hearts has been confirmed by studies of patient endomyocardial biopsies (5, 8, 38, 39). Additionally, histological staining identified neutrophils and lymphocytes (CD3^+^ T cells and CD20^+^ B cells) as part of the acute myocardial inflammatory infiltrate in TTS (8, 38–40). Unfortunately, attempts to further characterize these immune cells in biopsies are lacking, with only one study showing more M1-type pro-inflammatory macrophages in two TTS patient hearts compared to controls (8). Biopsies are not obtained routinely in the course of TTS, and so information on the long-term persistence of specific immune cells in the heart is currently unavailable. This line of inquiry may be better addressed using animal models, which will be reviewed later in this article.

As an alternative, researchers have examined circulating immune cells in TTS. One study found an increased percentage of classical CD14^++^CD16^−^ monocytes and a decreased percentage of intermediate CD14^++^CD16^+^ and non-classical CD14^+^CD16^++^ monocytes in the blood of TTS patients acutely, which resolved to normal levels at 5 months with the exception of the intermediate population that remained reduced (22). Classical monocytes are the most abundant of the three subsets in physiological circulation, characterized by their enhanced phagocytic ability, secretion of pro-inflammatory cytokines, and ability to differentiate into macrophages (41, 42). These monocytes may infiltrate the heart following myocardial injury in TTS, promoting inflammation and explaining the macrophage infiltrate seen in patients. Intermediate monocytes participate in antigen presentation and immune regulation (41), but because they are relatively understudied, it is difficult to imagine the potential contributions of their sustained decrease to TTS pathophysiology. Another study found that while circulating neutrophils in TTS patients release superoxides at a similar amount compared to controls upon *in vitro* stimulation, TTS neutrophils were apparently resistant to the suppression of superoxide release by B-type natriuretic peptide (43). This resistance was observed in neutrophils isolated from patients acutely and after a 3-month period, which could fuel the persistence of inflammation in TTS patients.

### 2.3 Summary

Overall, the available clinical literature suggests that TTS is characterized by a strong acute inflammatory response driven primarily by innate immune cells, which rapidly weakens into a mild, chronic inflammatory state. The importance of inflammation in determining TTS patient outcomes has so far been implied only by correlational analyses, or by the co-occurrence of chronic inflammation and poor outcomes at follow-up. However, there are reports of anti-inflammatory treatment being beneficial for patients. To our knowledge, four case reports on TTS patients treated with steroids have been published in the literature, with all patients having improved outcomes and normalized cardiac function after treatment. It should be noted though that all four had TTS in association with other conditions, namely microscopic polyangiitis (27), acute disseminated encephalomyelitis (44), allergy induced by dimethyl fumarate treatment for relapsing-remitting multiple sclerosis (45), and finally cytokine storm and LV outflow tract obstruction (32), which may have made the resulting inflammation more harmful. Incidentally, in addition to TTS being described in patients treated with immune checkpoint inhibitors (26) or diagnosed with underlying autoimmune rheumatic disease (46), inflammatory bowel disease (47), infection (including with COVID-19) (48, 49), and sepsis (25) among others, these cases bring to mind the perspective that inflammation may be a cause and not just a consequence of TTS. Due to their similarity in clinical presentation among other observations, myocarditis has also been suggested to trigger TTS (50), although this is under debate since myocarditis is typically considered a distinct clinical entity as well as an exclusion criterion for TTS.

## 3 Basic research

### 3.1 Rodent models of TTS

The rapid recovery of cardiac dysfunction and resolution of inflammation in most patients makes it difficult to study the dynamics of the acute immune response in TTS. The lack of available tissue samples at various points in the course of the disease also limits investigation into cardiac inflammation. Using animal models has been particularly helpful in overcoming these issues. Two rodent models of TTS are primarily used, employing either immobilization or catecholamine administration to the animal. The immobilization model involves restraining rats with tape to a board for specified periods of time. In this stress model, plasma catecholamine levels were increased as early as 15 min after immobilization and remained high up to 150 min later at the conclusion of the experiment in one report (51), and the induction of TTS-like cardiac phenotypes (e.g. apical ballooning) has been described (52). Unfortunately, the immune response has not yet been studied at length in these animals in the context of TTS, so we focus instead on the catecholamine administration model.

The second model involves the administration of a high catecholamine dose to animals, mimicking the catecholamine surge thought to be central to TTS pathophysiology. In most cases, the β1/2-adrenergic receptor agonist isoproterenol/isoprenaline (ISO) is injected intraperitoneally at doses greater than or equal to 100 mg/kg in mice or 5 mg/kg in rats (**Table 1**). The use of ISO aligns with evidence that β-adrenergic receptor activation may be responsible for the distinct cardiac phenotype in TTS (2). Mice or rats injected with a single high dose of ISO exhibit varying degrees of reversible cardiac dysfunction, apical ballooning, and regional wall motion abnormalities, similar to TTS patients (8, 18–20, 53–59, 62–66). How fast these phenotypes manifest after ISO administration varies but, depending on the study, they could completely resolve anywhere from as early as 6 hours to about 2 weeks post-ISO. However, there are reports where LV ejection fraction (LVEF) could improve but remain mildly reduced 4 weeks post-ISO (58), or where diastolic dysfunction could worsen 4 weeks post-ISO (65); LVEF could even increase post-ISO, with no TTS-like cardiac phenotypes observed, although this has only been observed in one report (61). Unlike patients, mortality rates can be especially high in the catecholamine model, with high-dose ISO-treated hearts showing considerable fibrosis and cardiomyocyte death (8, 18, 53–59, 62, 63, 65, 67). At this point, it should be emphasized that this model makes a key assumption regarding TTS, i.e., it is caused by a catecholamine surge, and as such cannot be expected to perfectly recapitulate the complexity of the disease in patients (68).

**Table 1:** Studies on the catecholamine (isoproterenol) animal model of TTS with findings related to the immune system.

Study	Animal	Sex*	ISO dose/s tested**	Route	Mortality	Troponin release	Cardiac function (LVEF)	Major immunological findings
53	C57BL/6J mouse	F	1x 300 mg/kg (injected 7 days after saline or 1x 300 mg/kg ISO)	i.p.	~25% over 7 days post-ISO in saline pre-treated group; 0% in ISO pre-treated group	Peak at 1 hr/day 1 post-ISO, minimal at day 7 post-ISO in saline pre-treated group; minimal at all time-points in ISO pre-treated group	Reduced at 1 hr post-ISO, normalized by 6 hr post-ISO onward in saline pre-treated group; normal at all time-points in ISO pre-treated group	A high dose of ISO dampened the immune response in the heart against a second, similar dose administered 7 days later; this cardioprotective effect was abolished when macrophages were depleted using clodronate liposomes
54/55	C57BL/6J mouse	F^†^	1x 100, 200, 300 mg/kg	i.p.	~15% over 7 days post-ISO	Peak at 1 hr post-ISO, high at day 1 post-ISO, minimal by day 3 post-ISO onward	Reduced at 1 hr post-ISO, normalized by 6 hr post-ISO onward	Brisk innate inflammatory response was induced by ISO, characterized by an initial influx of neutrophils, followed by monocytes, macrophages, and lymphocytes into the heart; also tested PD-1-KO mice, which showed an increased number of macrophages and CD4^+^/CD8^+^ T cells in the heart at day 7 post-ISO, and slower recovery of acute cardiac dysfunction
56	C57BL/6J mouse	F^†^	1x 200, 400 mg/kg	i.p.	0% over 7 days post-ISO	Similarly high at days 1 and 7 post-ISO	Reduced at day 1 post-ISO, normalized by day 3 post-ISO	Expansion of cardiac macrophages at day 1 post-ISO, which normalized at day 7 post-ISO (in both, Tim4^-^ infiltrating macrophages were predominant); depletion of macrophages using clodronate liposomes protected against ISO-induced cardiac dysfunction, with similar results using a CCR2 antagonist and Ccr2-KO mice, as well as inhibiting myeloid activation using Hif1α-KO mice and bortezomib
57	C57BL/6J mouse	n.i.	1x 160 mg/kg	i.p.	not studied	High at day 1 post-ISO	Reduced at weeks 2 and 4 post-ISO	Dendritic cells increased in number in the heart at week 1 post-ISO (mostly cDC1 cells), which decreased by weeks 2 and 4 post-ISO; also tested Clec9a-KO mice, which showed decreased cardiac immune infiltration at week 4 post-ISO, with improved cardiac function - these improvements may be linked with decreased CD8^+^ T cell activation
58	C57BL/6J mouse	M/F^††^	1x 40, 80, 160, 320 mg/kg	i.p.	Rare (<1% across doses over a year post-ISO)	High at day 1 post-ISO	Mildly reduced at week 2 post-ISO	Increased number of immune cells in the heart at week 1 post-ISO (CD11b^+^ myeloid and CD11c^+^ dendritic cells), normalized at week 2 post-ISO; increased CD4^+^ T cell activation in the heart (time-point not stated) and presence of anti-heart auto-antibodies at week 12 post-ISO suggest activation of a persistent autoimmune adaptive immune response
8	Sprague Dawley rat	F	1x 100 mg/kg	i.p.	10% following injection (time-point not indicated)	not studied	Mostly reduced at 6 hr post-ISO, remained reduced up to week 2 post-ISO	Focused on histopathology; immune infiltration in the heart as early as 6 hr post-ISO (neutrophils), peaked at days 3-4 post-ISO (macrophages), and diminished at day 5 post-ISO onward; T cells were not detectable in infiltrate; generally, M1 macrophages in the heart increased over time, wherease M2 macrophages increased, then decreased
59	Sprague Dawley rat	F	1x 5 mg/kg	i.p.	~40% within 2 hr post-ISO	not studied	Mildly reduced at day 1 post-ISO	Hearts primarily showed a macrophage/monocyte infiltrate at day 1 post-ISO; PARP-1 inhibition preserved ejection fraction at day 1 post-ISO, but had minimal effect on cardiac immune infiltration
60	Sprague Dawley rat	F	1x 150 mg/kg	i.p.	not studied	not studied	not studied	The expression of various Toll-like receptors (TLR2, TLR4, TLR6) showed dynamic changes post-ISO in cardiomyocytes and immune cells
61	C57BL/6J mouse	M	1x 5, 200, 300, 400, 500, 600, 700, 800, 1000 mg/kg	s.c.	0% following injection (time-point not indicated)	High at day 1 post-300 mg/kg ISO, but minimal for the 200 mg/kg dose	Increased at day 1 post-ISO (both doses) and day 3 post-ISO (200 mg/kg); normalized by week 1 post-ISO onward	Mild increase in proliferative inflammatory cells in hearts observed at week 1 post-ISO (both doses)
62	Sprague Dawley rat	M	1x 25, 50, 85, 100, 170, 200, 400, 600 mg/kg	s.c.	40% within 1 day post-ISO	not studied	EF not studied, but no significant differences in FS observed at day 1 post-ISO	Increased immune infiltration (neutrophils, monocytes) and edema in hearts at day 1 post-ISO, which was not ameliorated by pre-treatment with beta-blockers
63	Wistar rat	M	1x 5 mg/kg	s.c.	20% within 6 days post-ISO	High at day 1 post-ISO	Reduced at day 1 post-ISO, normalized at day 6 post-ISO	Increased immune infiltration in hearts at day 3 post-ISO; observed what was likely edema at days 1-3 post-ISO

n.i., not indicated; i.p., intraperitoneal; s.c., subcutaneous; ISO, isoproterenol; LVEF, left ventricular ejection fraction; FS, fractional shortening; KO, knock-out .

*main sex used in the study.

**main dose used in the study is highlighted in bold, and served as the basis for the rest of the information in the table.

^†^also performed some studies on the opposite sex (not detailed in the table).

^††^used M for mortality, troponin, and cardiac function data, and F for immunological studies.

### 3.2 Myocardial and systemic inflammation in the ISO model of TTS

**Figure 1:**
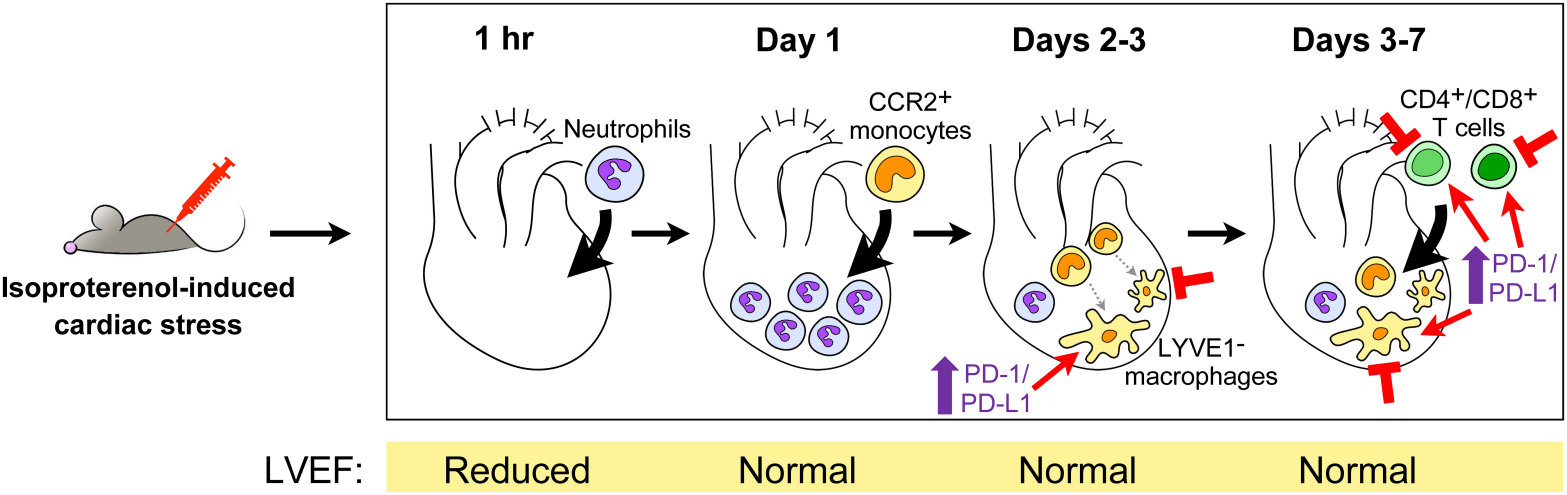
The cardiac inflammatory response in high dose isoproterenol-injected mice. Upon intraperitoneal injection of 300 mg/kg isoproterenol, mouse hearts undergo a series of changes in their immune cell landscape within the first 7 days following stress-induced injury (55). The accompanying changes in the left ventricular ejection fraction (LVEF) as a measure of cardiac function are shown below. Potential points of therapeutic intervention are indicated, i.e., depletion of macrophages or T cells, or the enhancement of programmed death-1 (PD-1)/PD-ligand 1 (PD-L1) signaling in macrophages and T cells. CCR, receptor for CCL2/MCP1 and CCL7/MCP3; LYVE1, tissue-resident macrophage marker.

Histologically, inflammatory infiltrates are observed in hearts by 2 days post-ISO in mice (55) or 6 hours post-ISO in rats (8), and could persist at milder levels at least up to 6 weeks later (58). Most recently, our group used flow cytometry to count the number of different immune cells in hearts within a 7-day period after intraperitoneally injecting mice with a single 300 mg/kg dose of ISO, to understand the temporal dynamics of the cardiac inflammatory response in this model (**Figure 1**) (55). The total number of cardiac CD45^+^ cells increased from baseline starting at day 2 post-ISO; this peaked at day 3 post-ISO, and then went down but remained slightly elevated after a week post-ISO. Ly6G^+^ neutrophil counts increased as early as an hour post-ISO, followed by an influx of Ly6C^high^CD64^low^ monocytes at day 1 post-ISO, and an elevated number of Ly6C^low-neg^CD64^+^ macrophages at day 3 post-ISO. In contrast to CD4^+^ T cells, which increased in number at day 3 post-ISO, CD8^+^ T cell and CD19^+^ B cell counts dropped at day 1 post-ISO and steadily increased thereafter. The counts of all these immune cell types eventually normalized with time but remained higher than baseline a week post-ISO, except for the CD19^+^ B cells whose number became comparable to baseline. These dynamics were confirmed by single-cell sequencing of CD45^+^-sorted cardiac leukocytes, revealing that LYVE1^−^ macrophages comprised the majority of CD45^+^ cells in the heart a week post-ISO. Immune cell trajectories were comparable in both sexes, with male hearts generally having a stronger inflammatory response. Where data is available for comparison, roughly similar findings have been shown in other studies, including in rats (8, 56, 57). In accordance with single-cell sequencing data, one group demonstrated that the number of CD11c^+^ dendritic cells was increased specifically in the murine heart a week after a single 160 mg/kg ISO injection, which suggests potential downstream activation of the adaptive immune response (57, 58). The conventional CD11b^−^XCR1^+^ dendritic cell population was expanded in particular, which is involved in the cross-presentation of endogenous antigens to CD8^+^ T cells.

### 3.3 Mechanistic studies

A few experiments have been performed to try and elucidate the role of inflammation in catecholamine-driven TTS pathogenesis. One report showed that high ISO dose administration in rats caused significant cardiomyocyte apoptosis 24 hr later, accompanied or followed by dynamic changes in the protein abundance of Toll-like receptors (TLRs) 2, 4 and 6 in cardiomyocytes and immune cells (60). In the setting of sterile injury, TLRs detect endogenous tissue antigens and subsequently activate inflammatory signaling (69, 70)—more thorough mechanistic studies could identify which TLRs and downstream signaling pathways are important in the immune response post-ISO. At the cellular level, depletion of monocytes and macrophages with clodronate liposomes preserved LVEF post-ISO in mice, with similar results when monocyte infiltration into the heart is prevented as in Ccr2-knockout mice, or mice treated with the CCR2 antagonist RS-504393 or the immunomodulatory drug bortezomib (56). Myeloid-specific knockout of Hif1α, a transcription factor involved in immune cell activation as well as adaptation to hypoxic conditions in sites of tissue injury, led to reduced numbers of neutrophils and macrophages in hearts post-ISO, which was accompanied by decreased levels of plasma cardiac troponin T and preserved LVEF (56). Although the specific contributions of resident cardiac macrophages were not clarified, the above findings highlight a key role for innate immune cells in catecholamine-mediated cardiac pathology.

On the other hand, our group revealed the importance of programmed death-1 (PD-1)/PD-ligand 1 (PD-L1) immune checkpoint signaling in ISO-induced acute myocardial injury (55). The PD-1/PD-L1 axis is perhaps best known for its functions in T cells, wherein the binding of PD-L1 ligand to the PD-1 receptor on T cells leads to the inhibition of T cell activity, a mechanism exploited by cancer cells to promote their survival (71). PD-1 and PD-L1 were acutely up-regulated in cardiac macrophages, CD4^+^ T cells, and CD8^+^ T cells post-ISO, with increased PD-L1 expression persisting up to a week post-ISO in macrophages and CD4^+^ T cells. Treating mice with anti-PD-L1 antibodies decreased survival and increased serum troponin I release post-ISO, as well as promoted immune infiltration into the heart. Interestingly, while wild-type and PD-1-knockout mice had a similar acute response to ISO-induced injury, PD-1 knockout mice had worse phenotypes later on with increased troponin leak and cardiac inflammation a week post-ISO. We also investigated the mechanism for the increased levels of PD-1 protein in myocardial immune cells after tissue injury and found that damage-associated molecular patterns released from necrotic myocardial cells provoked PD-1 up-regulation in a TLR2/TLR4/NF-κB-dependent manner in both innate and adaptive immune cells, e.g., macrophages, CD4^+^ T cells, and CD8^+^ T cells. The contributions of PD-L2, another ligand for PD-1, to ISO-driven cardiac pathology were also studied, but PD-L2 was only up-regulated in cardiac macrophages, CD4^+^ T cells and CD8^+^ T cells a week post-ISO and blocking it with an antibody did not produce significant differences in outcomes compared to controls.

Another group was interested on long-term phenotypes following ISO-induced cardiac injury, focusing on dendritic cells (58). Predominantly expressed in conventional dendritic cells, CLEC9A is a receptor whose activation by F-actin (released by dying cells) promotes the cross-priming of CD8^+^ T cells with necrotic antigens (72). *Clec9a*-knockout mice exhibited faster recovery than wild-type mice from the extended effects of ISO-induced injury, without affecting the number of leukocytes in the heart at baseline or a week post-ISO. In particular, *Clec9a*-knockout mouse hearts appeared to have normal histology by 4 weeks post-ISO and preserved LVEF by 2 weeks post-ISO, whereas these phenotypes could be observed up to 4 to 6 weeks post-ISO in wild-type mice (pathology was particularly persistent in the model used in this study). Hearts from *Clec9a*-knockout mice also had a reduced number of CD8^+^ T cells when evaluated at 4 weeks post-ISO. Corresponding with this data, *Cd8*-knockout mice had decreased cardiac fibrosis at 4 weeks post-ISO, suggesting that diminished CD8^+^ T cell activity due to deficient dendritic cell cross-priming drives the improved recovery found in *Clec9a*-knockout mice. The same group also interestingly performed adoptive transfer of total splenocytes from wild-type mice at 4 weeks post-ISO into recipient mice, which then developed a reduction in LVEF when evaluated 4 weeks later (57). Together with data showing an increased number of cardiac auto-antibodies in the serum of mice 12 weeks post-ISO (57), it is possible that ISO induces an auto-immune response against the heart that manifests later on in the course of ISO-mediated cardiac injury.

### 3.4 Studying TTS recurrence

As mentioned above, the recurrence of TTS is rare, and its pathogenesis is unknown. In our recent work, to investigate the role of the immune system in the setting of recurrent catecholamine-induced injury, we intraperitoneally administered a second 300 mg/kg dose of ISO 7 days after a first injection of ISO at the same dose. We interestingly observed no effects on cardiomyocyte death, cardiac inflammation, or changes in LV structure and function in ISO pre-treated mice compared to saline pre-treated controls (i.e., ISO-induced preconditioning) (53). Intriguingly, depletion of cardiac macrophages using clodronate liposomes at 7 and 9 days after the first ISO injection partially attenuated the cytoprotective effects against injury from the second ISO injection (administered 12 days following the first ISO dose). This result hints at a role for the immune system in establishing cardioprotection following catecholamine-induced injury to the heart, and suggests that TTS recurrence could, at least in part, be attributable to immunological mechanisms.

### 3.5 Summary

Taken together, data from the catecholamine animal model paint a picture of inflammation post-ISO akin to that seen in TTS patients, i.e., a strong, acute response driven by innate immune cells following the index event that resolves into a weaker state of persistent inflammation. Both the innate and adaptive immune systems appear to be implicated in persistent inflammation and may cause long-term pathology in patients at least in the context of catecholamine-driven TTS. We have only begun to understand which immune cells may be important contributors to catecholamine-driven cardiac pathology, but not so much how these cells perform such contribution. It is worth mentioning that significant LV dysfunction and troponin release coincide with the appearance of inflammation in most studies of the high-dose ISO model. There is currently no available data with sufficient resolution to determine whether inflammation occurs before or after the onset of these cardiac phenotypes—this could be a goal for future work. We showed in our report (55) that the peak of inflammation post-ISO happens well after the peak of cardiac troponin I release and the recovery of LVEF [similar to another study (8)], which could indicate a more critical role for inflammation in this model.

In contemplating the implications of findings from the catecholamine model, one must consider that this model exhibits a great deal of variability across studies, including in the kind and severity of outcomes observed as well as the rate of recovery post-ISO (**Table 1**). This could be influenced by multiple factors, e.g. the dose of ISO used, the route of ISO administration, the sex of the mice, the methods and time-points used for outcome evaluation, or even the way the mice are restrained when performing injections (54). Findings from one study where the model exhibits more persistent cardiac damage, dysfunction, or inflammation may therefore not necessarily apply to another where these phenotypes are more promptly resolved. Moreover, we only discussed a specific type of the catecholamine model here, i.e., the single high-dose ISO model, as it is the configuration of the model with the most wealth of information concerning inflammation. Other variations of the model do exist in the literature, such as those using two consecutive ISO doses instead of one (62, 73), or those using high doses of norepinephrine or epinephrine instead of ISO (17, 20), in which inflammation could act differently. Until a way to standardize the model has been developed, it is strongly recommended that the pathological context surrounding the catecholamine model used in a study be reviewed and considered when interpreting findings.

## 4 Conclusions and future directions

Accumulating mechanistic insights from clinical and basic research investigations consistently emphasize the association between inflammation and the pathogenesis, progression, and remission of TTS. While these studies have certainly given us insights into the sequence of events in TTS-driven inflammation, much remains to be understood regarding the immune cells that participate in this response. The signals that trigger the initial infiltration of immune cells into the heart following an index TTS event, the specific roles and fates of each immune cell type in the development and/or recovery of TTS pathology, the effects of a previous experience of inflammation on the recurrence of TTS, how the immune response affects non-immune cells including cardiomyocytes in TTS, the genetic factors that shape the TTS-associated immune response, whether variations in the immune response across patients explain the wide spectrum of TTS phenotypes found in the clinic, and how factors such as age, sex, and the presence of other comorbidities affect inflammation in TTS—these are only some of the outstanding questions that require further investigation. Information from such studies would not only help enrich our understanding of the immunology of TTS, but also offer starting platforms for translational research.

Although substantial uncertainties remain to be explored in clinical studies, the available experimental data encouragingly suggest that specific immune cells such as macrophages (53, 56) and dendritic cells (58) could serve as therapeutic targets for the treatment of TTS, as well as for preventing its recurrence. As a general approach, steroids might be a relatively safe therapeutic option to suppress both excessive innate and adaptive immune responses, with good outcomes in patients with TTS and ongoing inflammation, as described previously (27, 32, 44, 45). A recent study also demonstrated that calcineurin signaling was up-regulated in a sex-dependent manner in an epinephrine-induced mouse model of TTS, with the administration of calcineurin inhibitors/anti-inflammatory agents cyclosporine A or tacrolimus leading to less severe symptoms (74). This suggests that anti-inflammatory therapies other than steroids may be beneficial to TTS patients. The available knowledge we currently have on TTS pathogenesis supports the possibility of risk-stratifying TTS patients according to their myocardial and systemic inflammation profiles, which could be non-invasively evaluated using well-established methods such as CMR imaging and serum CRP level detection, respectively (22, 23). Such an assessment would enable the identification of patients who might benefit from anti-inflammatory treatment. Moving forward, research efforts using novel technologies such as longitudinal single-cell sequencing analysis or other high-resolution immune-profiling methods that enable researchers to comprehensively test the hypothesis that immune cells play a critical role in TTS will be needed to expand our understanding on the role of inflammation in this disease, as well as to develop more rational, mechanism-based therapies, e.g. biologics and monoclonal antibodies targeting specific immune cell types or cytokines involved in TTS.

## Author contributions

KL: Writing – original draft, Writing – review & editing. DM: Writing – review & editing, Supervision. TK: Supervision, Writing – review & editing. TH: Supervision, Writing – review & editing, Conceptualization, Project administration, Writing – original draft.
